# Pentraxin-3 inhibits milky spots metastasis of gastric cancer by inhibiting M2 macrophage polarization

**DOI:** 10.7150/jca.58698

**Published:** 2021-06-04

**Authors:** Xinye Cui, Tao Qin, Zhengdong Zhao, Guang Yang, Jaceline Gislaine Pires Sanches, Qingqing Zhang, Shujun Fan, Liang Cao, Xiang Hu

**Affiliations:** 1 Department of General Surgery, The First Affiliated Hospital, Dalian Medical University, Dalian 116011, P.R. China.; 2 Department of Oncology, Qingdao Municipal Hospital, School of Medicine, Qingdao University, Qingdao, Shandong 266071, P. R. China.; 3 Department of General Surgery, The Second Affiliated Hospital, Dalian Medical University, Dalian 116027, P.R. China.; 4 Department of Pathology, Dalian Medical University, Dalian 116044, P. R. China.

**Keywords:** PTX3, Gastric cancer, Milky spot, Macrophage polarization

## Abstract

**Purpose:** Recent studies have indicated that Pentraxin-3 (PTX3) is related to invasion, migration and metastasis of gastric cancer cells (GCCs). However, the function of PTX3 in stemness and tumor-associated macrophages (TAMs) polarization in GC has not yet been revealed. Here, we investigated the role of PTX3 in TAMs polarization and stemness in gastric cancer (GC), and further explored the effect of PTX3 on milky spot metastasis of gastric cancer.

**Methods:** PTX3 expression in human gastric cancer tissues was examined with immunohistochemistry (IHC). The influence on stemness of gastric cancer cells was examined by sphere formation assay and western blot. qRT-PCR, IHC and flow cytometry were used to evaluate M1/M2 macrophage signatures. The effects of PTX3 on TAM polarization and milky spots were investigated *in vitro* and *in vivo*. The possible mechanism of PTX3 on targeted cytokines and pathway were analyzed by qRT-PCR and western blot.

**Results:** We found that PTX3 was low expressed in gastric carcinoma tissues and associated with stemness and polarization of macrophages. The upregulation of PTX3 inhibited the stemness of GCCs. Furthermore, PTX3 suppressed the polarization of M2 macrophages in the milky spots *in vivo* and *in vitro* and inhibited the metastasis of GC into milky spots. PTX3 restrained the expression of interleukin-4 (IL-4) and IL-10 via the inhibition of phosphorylation of the c-Jun N-terminal protein kinase 1/2 (JNK1/2) in GCCs.

**Conclusion:** These results revealed a novel mechanism of PTX3 in GC, which may participate in the development and metastasis of GC by affecting stemness and macrophage polarization. PTX3 should be considered as a crucial biomarker and may be potentially used in targeted therapy in GC progression.

## Introduction

Gastric cancer (GC) is a life-threatening malignant tumor in humans. It is the fifth most common cancer and the second leading cause of cancer-related mortality globally 1, 2, with high recurrence and low survival rates, especially in patients with advanced forms. The prevention and treatment of GC are greatly challenged by cancer regeneration and metastasis. The tumor microenvironment (TME) is formed due to the interaction between tumor cells and the host. This unique environment is formed and dominated by tumors, which monitor molecular and cellular events occurring in the surrounding tissues 3. Tumor progression is regulated by the crosstalk between cancer cells and other cytokines in the TME with tumor-infiltrating myeloid cells possibly having an important role in immunosuppression 4. Tumor-associated macrophages (TAMs) play an important leading role in tumor-related inflammation. Recent evidence indicates that as a vital regulator of tumorigenesis, these cells have dual pro- and anti-tumor activity 5, 6. In addition, data from various clinical and experimental studies in recent years have shown that TAMs promote the progression and metastasis of solid tumors by releasing a variety of cytokines, including chemokines, inflammatory factors and growth factors 7-9. TAMs are mainly manifested in two subtypes, namely M1 (classically activated macrophages), which are polarized by lipopolysaccharide (LPS) and interferon-γ (IFN-γ) and M2 (alternatively activated macrophages), which are polarized in the presence of interleukin-4 (IL-4), IL-10 or IL-13. These two cell types usually have opposite effects on tumor progression 10. Studies in recent years have proved that in malignant tumors, macrophages mainly express an M2-like phenotype 11. M2 macrophages have immunosuppressive effects that are conducive for angiogenesis and tissue repair 12 but are contrary to the effects of pro-inflammatory and cytotoxic M1 cell expression. In addition, studies have shown that tumor-related M2 macrophages can improve tumor cell growth and survival and stimulate angiogenesis and tumor metastasis 13.

Peritoneal dissemination is still one of the most common patterns of metastases, which is a severe issue with a significant impact on the survival rate of patients with GC. Since milky spots are the main implantation sites of tumor cells in peritoneal transmission, researches on the mechanisms of peritoneal dissemination have been mainly focused on the milky spot. Milky spots are primitive lymphoid tissues in the abdominal cavity of humans and animals, mainly in the greater omentum, mesentery and pelvic floor. It is worth noting that the milky spot is a tiny anatomical structure, mainly composed of massive macrophages and lymphocytes and participates in the removal of peritoneal cavity particles, bacteria and tumor cells 14. The milky spot is a common metastasis site for ovarian, colon and stomach cancer. More importantly, peritoneal milky spot macrophages are the first line of defense of the peritoneum. Studies have shown that GCCs preferentially and selectively infiltrate milky spots during the spread of peritoneal cancer, thereby forming micrometastasis 15. Therefore, it is essential to elucidate the interaction between the metastasis of GC into a milky spot and macrophage polarization.

Pentraxin-3 (PTX3) is a prototype member of the long-pentraxin subfamily, which is produced by innate immune cells and plays a vital role in inflammation regulation, innate immunity, as well as in tumor-related inflammation, including tumor occurrence, angiogenesis, metastasis and tumor immune regulation 16. Since PTX3 may affect many types of tumor cells, it is essential to clarify the biological role of PTX3 in tumor development. In our previous studies, we have demonstrated the differences in the expression of PTX3 between GCCs and normal gastric mucosal epithelial cells and GC tissues and adjacent tissues and proved the role of PTX3 in the epithelial-mesenchymal transition (EMT) process of GCCs 17. EMT plays an important role in the formation and metastasis of GC because GC stem cells have high invasiveness and EMT properties. Numerous pieces of evidence show that there is a connection between stem cells and EMT in GC 18. In the present study, we further investigate the role of PTX3 in the progression and metastasis of GC, demonstrate the correlation between PTX3 and GC stemness and related effects on the polarization of macrophages in milky spots metastasis. Moreover, we clarify the mechanism of metastasis into milky spots.

## Materials and methods

### Collection of human GC samples

All human gastric cancer tissues were obtained from tissue arrays, which were purchased from Shanghai OutdoBiotech (Shanghai, China). For further test of PTX3 expression, there included 30 cancer tissue samples and 30 paracancerous tissue samples. For the detection of the link between PTX3 and macrophage expression, the tissue arrays included 40 tumor tissues and adjacent tissues. They will be used for immunohistochemical analysis. All the patients were gastric cancer patients who underwent radical operation for carcinoma of stomach. The diagnosis of gastric cancer was confirmed by pathology, and no distant metastasis in all patients.

### Cell culture and transient transfection

Human GC cell lines (BGC-823, SGC-7901), human monocyto-macrophage line (THP-1) and strain 615 murine-derived GC cells (MFCs) were all obtained from the Laboratory of Pathology in Dalian Medical University in 2018, which have been tested and authenticated. BGC-823 cells were cultivated with 10% fetal bovine serum (FBS) in Dulbecco's Modified Eagle's medium (DMEM) (GIBCO, USA). SGC-7901, MFCs and THP-1 cells were cultivated in Roswell Park Memorial Institute (RPMI) 1640 medium (Gibco, USA) complemented with 10% FBS. Cultures were incubated at 37 °C with a 5% CO_2_ atmosphere. For transient transfection, the two GCCs were transfected with a plasmid using Lipofectamine 3000 and Opti-MEM (Invitrogen) following the product instructions.

### Macrophage differentiation and cell co-culture

THP-1 cells were used to generate and differentiate macrophage. Briefly, THP-1 cells were differentiated into macrophages (M0 macrophage) using 320 ng/mL phorbol 12-myristate 13-acetate (PMA, Abcam, ab120297) for 24 h. For M2 macrophages differentiation, THP-1 cells were first treated with 320 nM PMA for 24 h and cultured with 20 ng/ml IL-4 and 20 ng/ml IL-13 (Prime Gene, Shanghai, China) for 48 h. Macrophages were also co-cultured with GC cell lines (BGC-823, SGC-7901). GCCs were seeded in the upper insert of a six-well Transwell apparatus (0.4 μm pore size, Jet, Guangzhou, China), while macrophages were seeded in the lower chamber. After 48 h of co-culture, both GCCs and differentiated macrophages were harvested for further experiments.

### Plasmids, lentivirus and reagents

PTX3 overexpressing plasmid was produced by cloning PTX3 cDNA into the pcDNA3.1 vector (GenePharma, Suzhou, China). The two GC cell lines were transfected with a PTX3overexpressing plasmid (PTX3) and a control plasmid (CTL); the procedure followed the Lipofectamine 3000 system. Lentivirus vectors containing LV-PTX3 overexpressing PTX3 and the negative control (Scramble) were purchased from GenePharma Company (Suzhou, China). Puromycin (Clontech, USA) was used to treat these transfected cells. Recombinant human Pentraxin-3 protein was acquired from the R&D system (USA).

### Quantitative reverse transcription-polymerase chain reaction (qRT-PCR)

Total RNA from cultivated cell lines was extracted using Trizol reagent (Thermo, USA). Briefly, 1 μg of total RNA was reverse-transcribed using the cDNA Synthesis SuperMiX kit (Transgen, China). Messenger RNA (mRNA) expression levels of PTX3, IL-4, IL-10, TNF-α, CCR7, CXCL9, IL-1β, CCL22, TGF-β and ARG1 were measured with RT-PCR, which was analyzed by the iCycler™ Real-Time System and a SYBR Premix EX Tag Master mixture kit (Transgen, China) following the product instructions. The 2^-ΔΔCt^ method was used to evaluate mRNA relative expression levels. The gene sequences for qRT-PCR are as shown in Table 1.

### Western blot analysis

The whole-cell lysis assay kit (KeyGEN BioTECH, China) was used to treat the transfected cells after washing with cold phosphate-buffered saline (PBS). Protein concentrations were measured with the Easy II Protein Quantitative Kit (Transgen, China). Equal quantities of protein were transferred to the polyvinylidene difluoride (PVDF) membrane (Millipore, USA) after separating it on 12% sodium dodecyl sulfate-polyacrylamide gel electrophoresis (SDS-PAGE). The PVDF membranes with protein were blocked by 5% skimmed milk at room temperature for 2 h and then hatched with the specific antibody overnight at 4 °C. Secondary antibodies were then incubated with the transfer membranes. Finally, the images of the bands were captured with the ODYSSEY infrared imaging system. Specific primary antibodies against PTX3, GAPDH, IL-4, IL-10, SOX2, ALDH1, CD44 and CD133 were purchased from Proteintech Group, Inc. (Wuhan, China). Anti-LGR5 was obtained from Bioworld Technology, Inc. (Nanjing, China), anti-JNK1/2 and anti-p-JNK1/2 were purchased from Beyotime (Shanghai, China) and secondary antibodies were obtained from Santa Cruz Biotechnology (USA).

### Migration and invasion experiment

The same amount of the two GCCs (BGC-823 and SGC-7901) were co-cultured with M2 macrophages or rhPTX3-treated-M2 macrophages for 24 h. In the migration experiment, 1 × 10^5^ cells without serum were seeded into upper chambers (Corning, USA). A medium with 20% serum was added into the bottom chamber. Then, the upper cells were cleaned with cotton swabs 12-16 h later. Cells were then stained with 1% crystal violet and analyzed by an optical microscope. In the invasion assay, the same amount of cells without serum were seeded into the upper chambers (Corning, USA) and pretreated with Matrigel (BD, USA). The subsequent experimental steps were performed as in the migration experiment.

### Cell immunofluorescence

Approximately 3 × 10^3^ cells were counted and seeded in 24-well plates and then fixed with 4% paraformaldehyde. Subsequently, cells were washed with PBST (PBS and 0.5% Triton X-100) and blocked using 3% bovine serum albumin (BSA) at 4°C for 2 h. Next, cells were incubated with primary antibodies (Proteintech Group, Inc. China) overnight at 4°C: CD86, 1:100, CD206, 1:100. Afterward, fluorescence-conjugated secondary antibodies were added and incubated at 37 °C for 1 h. The cell nuclei were stained with the DAPI (4',6-Diamidino-2-phenylindole dihydrochloride) staining reagent and cells were viewed using the CKX41 Inverted Microscope (Olympus, Japan).

### Sphere formation assay

The GCCs (BGC-823 and SGC-7901) were counted to 1000/well and added into a low adhesion six-well plate (Corning, New York, USA). B27 (1:50, Invitrogen) was supplemented in each well of the plate containing DMEM/RPMI-1640 medium, human recombinant EGF (20 ng/ml) (Sigma-Aldrich, St. Louis, Missouri, USA) and bFGF (20 ng/ml) (Sigma-Aldrich, St. Louis, Missouri, USA). The number of colonies formed was calculated using an inverted microscope (Olympus Corporation, Tokyo, Japan).

### Flow cytometry

Phorbol 12-myristate 13-acetate (PMA)-treated THP-1 monocytes were co-cultured with GCCs for 48 h, and then the macrophages were collected for flow cytology analysis. The cell suspension was collected and washed twice using cold PBS. Cells were then stained with CD206-PE (BD Pharmingen, #555954) and CD86-APC (BioLegend, #374208). Meanwhile, Cytofix/Cytoperm Soln Kit (BD Biosciences, #554714) was used to fix and permeate the macrophages following the manufacturer's instructions. In addition, all detections were regulated by isotype control antibodies: PE mouse IgG1 κ isotype control (BD Pharmingen, #555749) and APC Mouse IgG1 κ Isotype Control RUO (BD Pharmingen, #554681).

### *In vivo* experiments

Animal experiments were conducted following the National Institutes of Health (NIH) guidelines for the Care and Use of Laboratory Animals and were approved by the Animal Care Committee of the Dalian Medical University. Ten six-week-old 615 strain mice were randomly separated into two groups, which were raised under standard laboratory conditions. LV-PTX3-transfected MFC cells (LV-PTX3) and negative control cells (Scramble) (5 × 10^5^) in 100 μL PBS were inoculated into the abdominal cavity of the animals. The mice were euthanized on the 14^th^ days respectively. The omenta of each mouse were then extracted and compared.

### Immunohistochemistry

Paraffin-embedded tissues were cut into slices about 5 μm thick and immobilized onto glass slides. Tissue sections were pre-incubated with 10% normal goat serum and subsequently incubated with primary antibodies overnight at 4 °C. Tissue slices were hatched with the secondary antibody for 10 min at 37 °C, washed with cold PBS, and then treated with peroxidase-conjugated biotin-streptavidin complex for 10 min, followed by staining with DAB (3, 3'-Diaminobenzidine) and hematoxylin. Staining extent was scored as follows: positive cell number ≤ 10%, 0 points; 11- 50%, 1 point; 51- 75%, 2 points; ≥ 76%, 3 points. The intensity of the staining was as follows: 0 = negative, 1-2 points = weakly positive, 3-4 = positive, >5 points = strongly positive. Immunostained tissues were evaluated using the intensity and extent of staining score.

### Statistical analysis

Data are presented as mean ± standard deviation (SD). Data were analyzed using GraphPad Prism 7 software. The Student's t-test was used to compare the control and the treatment groups. *P-*value <0.05 was considered statistically significant.

## Results

### PTX3 is low expressed and correlates with low M2 macrophage content in human GC tissues

**Figure 1:**
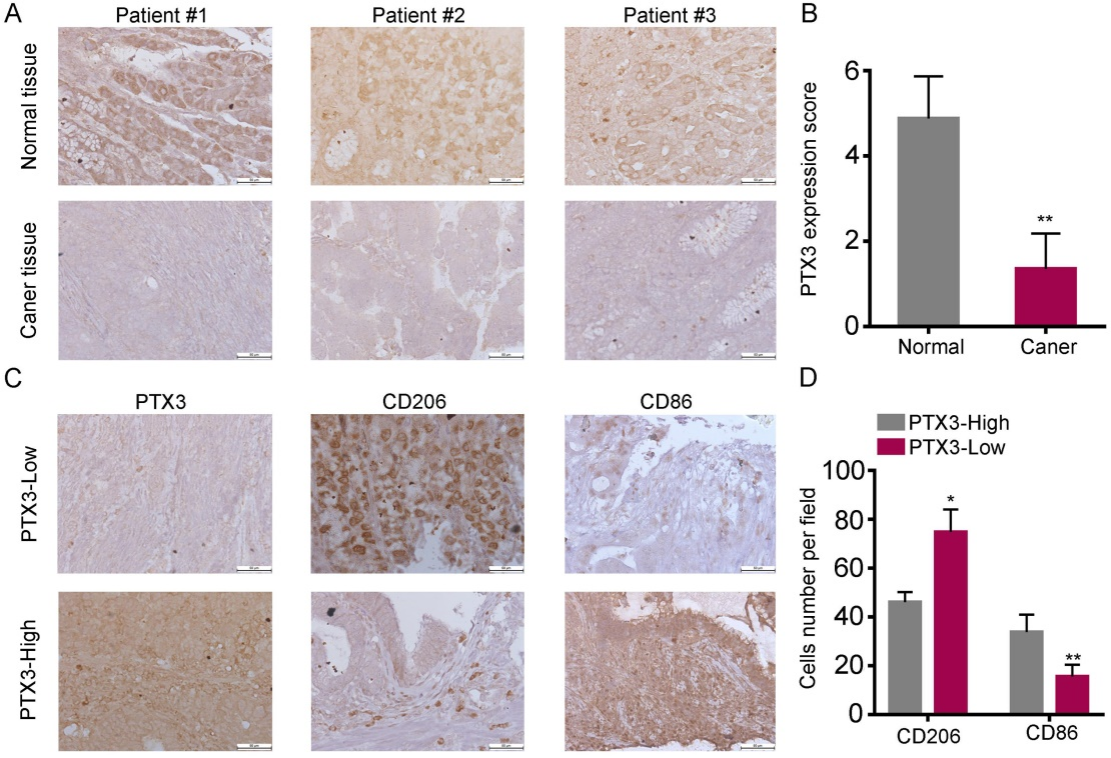
Low expression of PTX3 in human gastric cancer tissues and is associated with high M2/low M1 content. (A) PTX3 expression level in gastric cancer tissues and surrounding tissues were shown by immunohistochemistry (IHC). (B) PTX3 IHC staining scores in gastric cancer tissues (n = 30) and surrounding tissues (n = 30) were shown. (C) The protein expression of PTX3, CD206, CD86 in a human gastric cancer tissue array was tested by IHC. (D) Number of CD206+ and CD86+ cells per field in tissues with different levels of PTX3 expression. Representative pictures of IHC were shown (Scale bars: 50 μm) (*P < 0.05, **P < 0.01).

In our previous experiments, the high expression of PTX3 in normal gastric mucosal epithelial cells and adjacent cancer tissues was demonstrated via qRT-PCR and western blot 17. In this research, we used the tissue arrays including 30 cancerous tissues and para-cancerous tissues, and added the IHC analysis for the difference of PTX3 expression as a supplement and found similar results (Fig. 1A, B), which showed that PTX3 was higher expressed in normal tissues compared with GC tissues. Evidence from clinical and experimental studies has indicated that TAMs promote the invasion and metastasis of solid tumors by releasing a variety of cytokines. We have proven through previous studies that PTX3 can affect chemokine to inhibit the invasion and metastasis of GCCs 17. Therefore, we speculated whether PTX3 is linked to macrophages. Subsequently, we analyzed the correlation through IHC tests using tissue arrays. To quantify and distinguish the phenotype of macrophages, the expression of CD86 (M1 marker) and CD206 (M2 marker) were detected. As shown in Fig. 1C, D, there was significantly more infiltration of CD86+ cells than CD206+ cells in tissues with high PTX3 expression, while CD206+ macrophages showed significantly increased infiltration than CD86+ cells in tissues with low PTX3 expression. These results indicated that the expression of PTX3 in human GC tissues might affect the differentiation of macrophages.

### Upregulation of PTX3 inhibits GC cell stemness

**Figure 2:**
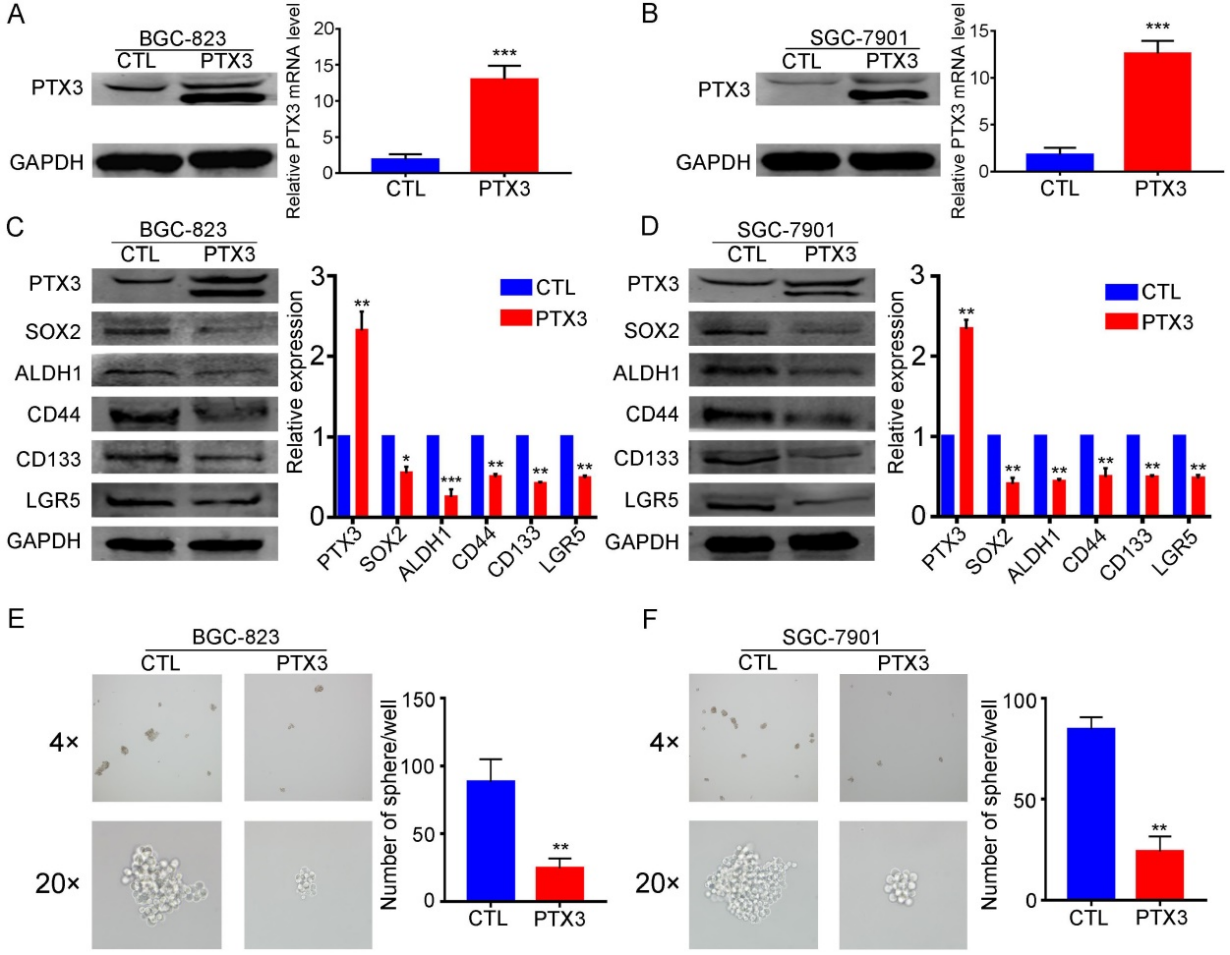
Overexpression PTX3 inhibits gastric cancer cell stemness and sphere formation ability. (A, B) BGC-823 and SGC-7901 cells were transfected with PTX3 overexpression plasmid (PTX3) or a negative control plasmid (CTL). PTX3 upregulation efficiency was confirmed by Western blotting and qRT-PCR. (C, D) The protein expressions of the CSCs markers SOX2, ALDH1, CD44, CD133 and LGR5 were detected in BGC-823-PTX3 or BGC-823-CTL cells, SGC-7901-PTX3 or SGC-7901-CTL cells by western blotting. (E, F) Representative photos of sphere formation ability after upgrading PTX3 in BGC-823 and SGC-7901 cells (original magnification, 4×, 20×) (*P < 0.05, **P < 0.01, ***P < 0.001).

Accumulating evidence shows that EMT and cancer stem cell (CSC) phenotypes are largely related, providing characteristics of invasion, tumor seeding, drug resistance and survival. CSCs in the primary tumor are thought to achieve distant metastasis through the EMT process 19. Furthermore, the CSC phenotype can be obtained by inducing the tumor EMT state 20. In our previous studies, we have confirmed that overexpression of PTX3 can inhibit the EMT process in GCCs (BGC-823 and SGC-7901) 17. However, we have not yet proved whether PTX3 affects the stemness of GCCs. In this study, we investigated whether overexpression of PTX3 inhibits cell stemness in GCCs. The expression of CSC markers (SOX2, ALDH1, CD44, CD133 and LGR5) 21, 22 in GCCs was determined by western blotting. We found that the upregulation of PTX3 reduced the expression of SOX2, ALDH1, CD44, CD133 and LGR5 (Fig. 2C, D). In addition, we further validated these results through a sphere-forming assay in which the sphere formation ability in the PTX3-overexpressed group was weaker than that in the control group (Fig. 2E, F). Therefore, the quantity and size of spheres were smaller in the experimental group than those in the control group. This indicated that PTX3 overexpression was involved in the attenuation of stemness and inhibited the ability to form spheres in the GCCs.

### Recombinant PTX3 inhibits M2 macrophages polarization and attenuates M2 macrophage-mediated gastric carcinoma progression *in vitro*

**Figure 3:**
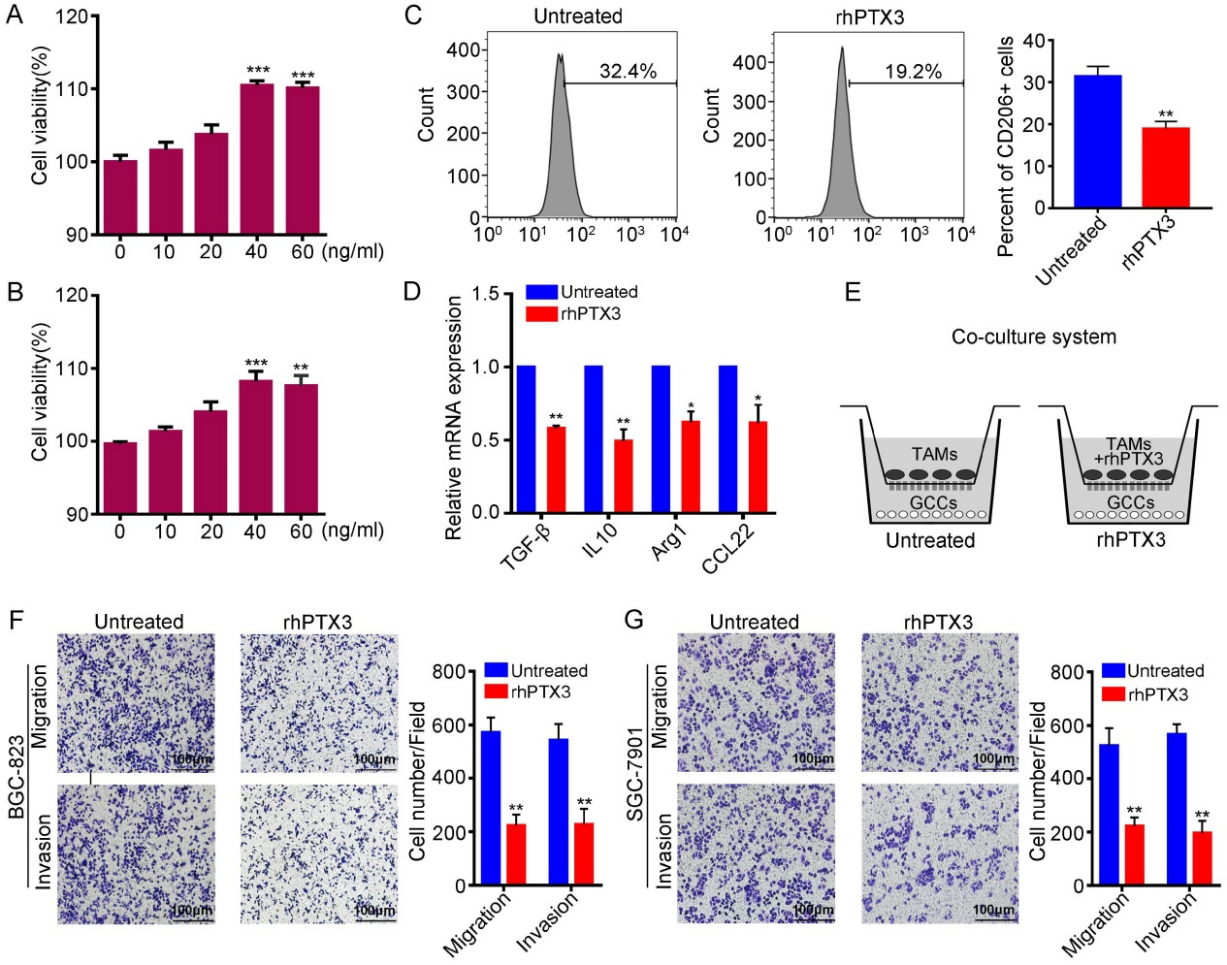
Recombinant PTX3 inhibits M2 macrophages polarization and suppresses gastric cancer progression promoted by M2 macrophages. (A) The viability assay of rhPTX3 with different concentrations (0 to 60 ng/ml) on the THP-1 cells through the CCK-8 assay for 48 h. (B) The viability assay of rhPTX3 with different concentrations (0 to 60 ng/ml) on the THP-1 cells through the CCK-8 assay for 72 h. (C) THP-1 cells were treated with 320 nM PMA for 24h, then cultured by the additional IL4 and IL13 with or without rhPTX3 (40 ng/ml) for another 48 h. Detection of CD206 expression by flow cytometry. (D) THP-1 cells were treated as indicated in (C), gene expressions of Arg1, IL10, TGF-β and CCL22 were examined through qRT-PCR. (E) Schematic diagram of co-culture system for TAMs and gastric cancer cells. (F, G) Representative images from cell migration and invasion assay of BGC-823 and SGC-7901 cells after co-culturing with M2 macrophages (Untreated) or rhPTX3-treated-M2 macrophages ( rhPTX3) (Scale bars: 100 µm) (*P < 0.05, **P < 0.01).

It is known that M2 macrophages can enhance angiogenesis and tissue remodeling, suppress anti-tumor immune responses and promote cancer metastasis 13. Therefore, macrophage research focuses more on M2 differentiation. To explore the effect of PTX3 on M2 macrophage polarization, we applied recombinant human Pentraxin-3 (rhPTX3). First, we performed the viability assay of rhPTX3 on the THP-1 cells. Cells were exposed to serial concentrations of rhPTX3 from 0 to 60 ng/ml for 48 h or 72 h. The cell inhibitory activities were measured using the Cell Counting Kit-8 (CCK8) assays and the results are shown in Fig. 3A, B. There was no significant growth inhibition of recombinant PTX3 on THP-1 cells. Subsequently, we analyzed the effect of rhPTX3 on M2 macrophages polarization induced by IL-13 and IL-4 *in vitro*. THP-1 cells were treated with 320 nM PMA for 24 h and cultured with IL-4 and IL-13 without (Untreated) or with rhPTX3 (40 ng/ml) for another 48 h. We found that the expression of CD206 of macrophages was significantly reduced in the rhPTX3 group through flow cytometry (Fig. 3C). To further demonstrate the effect of PTX3 on the polarization of M2 macrophages, the transcriptional changes of specific M2 marker genes were evaluated by qRT-PCR. The mRNA levels of M2 markers, Arg1, IL-10, TGF-β and CCL22, were all decreased upon treatment with rhPTX3 compared to the IL-13, IL-4-treated group (Fig. 3D). Moreover, we co-cultured M2 macrophages (Untreated) or rhPTX3-treated-M2 macrophages (rhPTX3) with GCCs to explore the impact of PTX3 on migration and invasion ability of GCCs mediated by M2 macrophages. We found that the migration and invasion capacity of GCCs mediated by M2 macrophages were decreased in the rhPTX3 group (Fig. 3F, G). Overall, these results suggested that PTX3 could inhibit macrophage M2-like polarization and alleviate gastric carcinoma progression mediated by M2 macrophage.

### PTX3 suppresses the polarization of M2 macrophages under mimic milky spot metastasis environment *in vitro*

**Figure 4:**
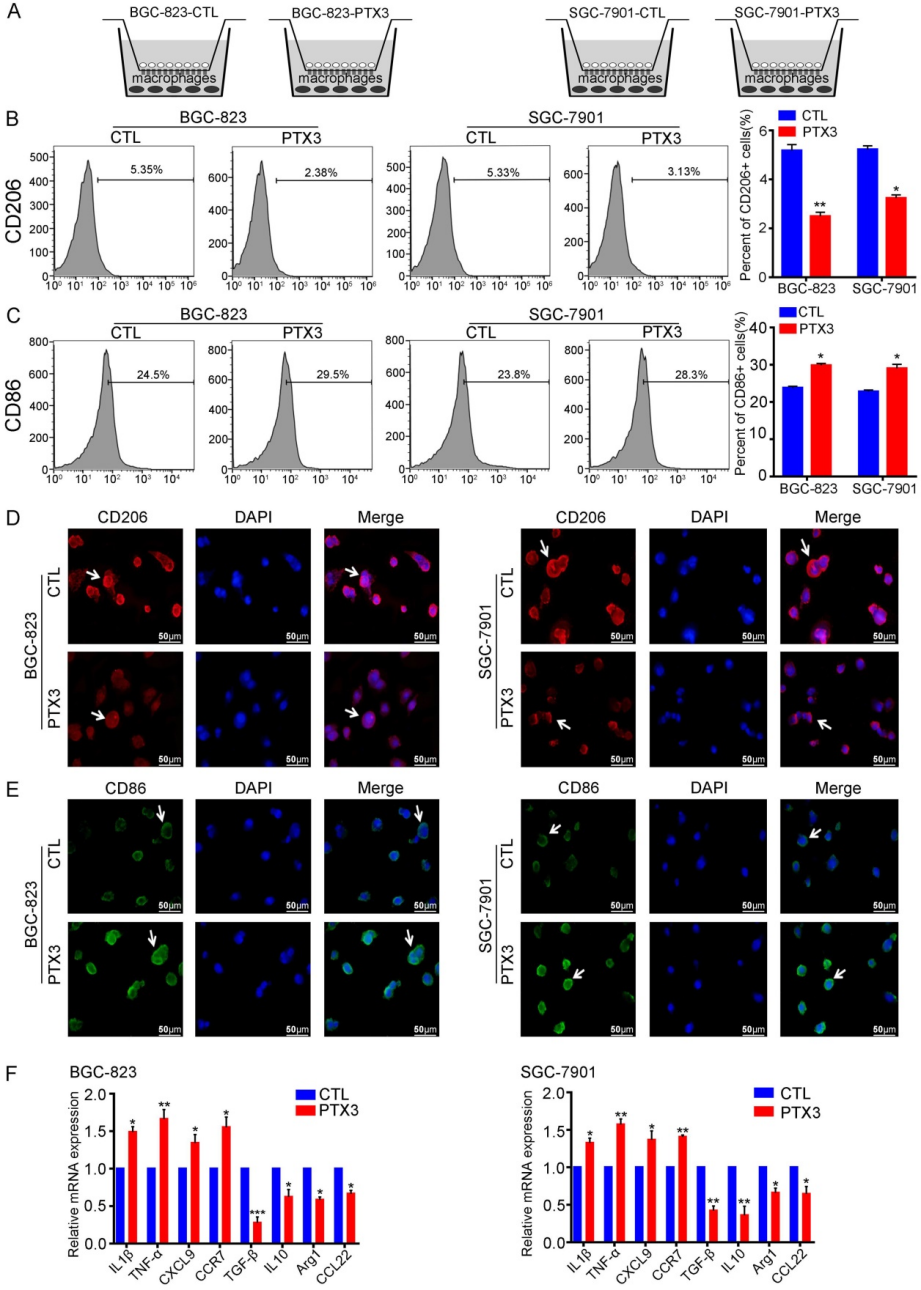
PTX3 in gastric cancer cells reduces M2 macrophages polarization and increases M1 macrophages polarization *in vitro*. (A) The co-culture system of gastric cancer cells and macrophages which mimics the environment of milky spots metastasis. (B, C) BGC-823 and SGC-7901 cells were transfected with PTX3 overexpression plasmid (PTX3) or a negative control plasmid (CTL), then co-cultured with PMA-treated macrophages. The expressions of CD206 and CD86 were analyzed by flow cytometry. (D, E) The two gastric cancer cells and macrophages were treated as indicated in (B, C), the expressions of CD206 and CD86 in macrophages were detected by Immunofluorescence staining (Scale bars: 50μm) (Arrows point the cells of interest). (F) Gastric cancer cells and macrophages were treated as indicated in (B, C), the mRNA expressions of M1 markers (IL1β, TNF-α, CXCL9, CCR7) and M2 markers (Arg1, IL10, TGF-β and CCL22) was investigated by qRT-PCR (*P < 0.05, **P < 0.01, ***P < 0.001).

The milky spot is a small specific structure composed of large macrophages, which is a common peritoneal metastasis site for the stomach, colorectum, and ovary cancers 23. To investigate the impact of PTX3 on the polarization of macrophages in the GC milky spot metastasis environment, we co-cultured GCCs with macrophages to simulate the GC metastasis environment into milky spots (Fig. 4A). We divided the co-cultured systems into two groups in each GC cell line, one of which PTX3 was overexpressed. Immunofluorescence and flow cytometry results showed that overexpression of PTX3 in BGC-823 and SGC-7901 cells decreased CD206 expression in macrophages compared with the control groups (Fig. 4B, D). Besides, the expression of the M1 macrophage marker, CD86, was also evaluated. Interestingly, CD86 expression in macrophages increased relatively in the co-cultured system with PTX3 upregulation (Fig. 4C, E). To further investigate these macrophage phenotypes, gene expression levels of M1 markers (IL-1β, TNF-α, CXCL9 and CCR7) and M2 markers (Arg1, IL-10, TGF-β and CCL22) was examined. A similar transcription pattern for macrophage markers was observed (Fig. 4F). There was a higher expression of IL-1β, TNF-α, CXCL9 and CCR7 and a lower expression of Arg1, IL-10, TGF-β and CCL22. Taken together, these results imply that under the condition of co-cultured systems, PTX3 could decrease the polarization of M2 macrophages while increasing the polarization of M1 macrophages.

### PTX3 restrains M2 polarization by suppressing the expression of IL-4 and IL-10 via negative regulation of JNK1/2 in GC cells

**Figure 5:**
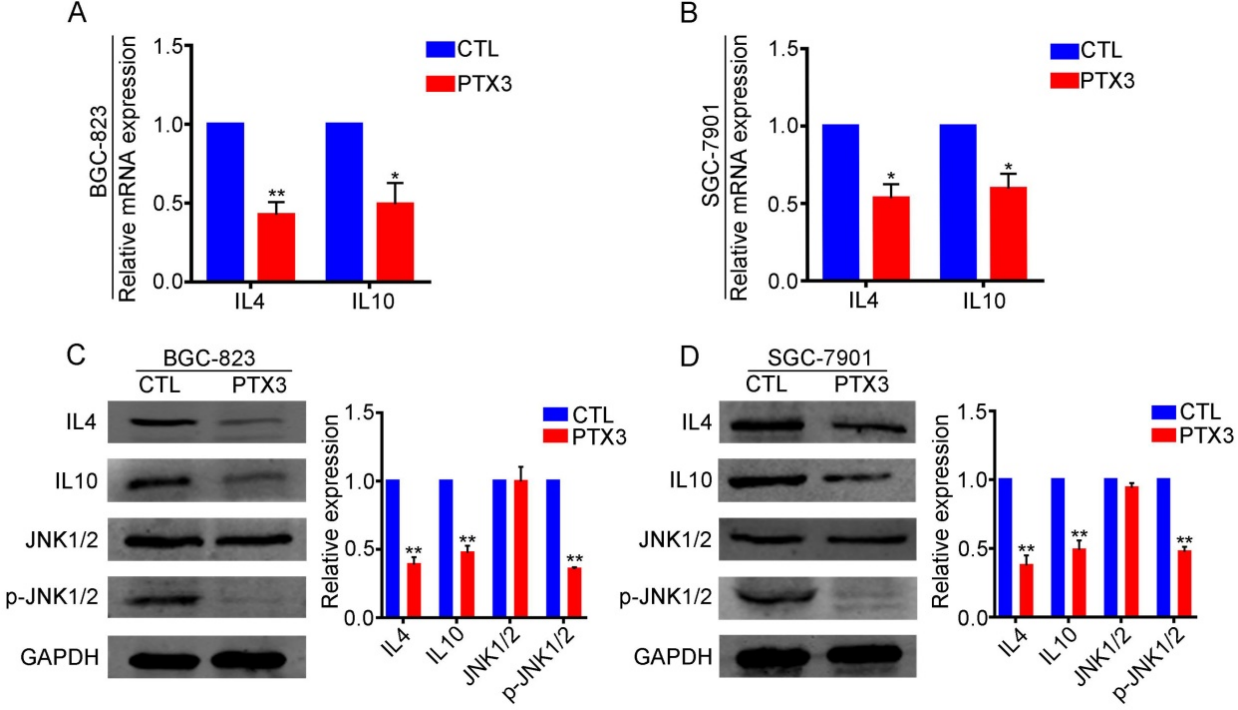
PTX3 negatively regulates IL4 and IL10 expression via inhibiting JNK1/2 phosphorylation in gastric cancer cells. (A, B) The mRNA levels of IL4 and IL10 in BGC-823 and SGC-7901 cells after upregulating PTX3 were checked by qRT-PCR. (C, D) BGC-823 and SGC-7901 cells were transfected with PTX3 overexpression plasmid (PTX3) and a negative control plasmid (CTL). Detection of IL4, IL10, JNK1/2 and p-JNK1/2 via western blot assay (*P < 0.05, **P < 0.01).

M2 macrophage polarization depends on the presence of IL-4 or IL-10. Therefore, we speculated that PTX3 might suppress M2 polarization through the regulation of IL-4 and IL-10 expression in GCCs. Based on this conjecture, we performed qRT-PCR and western blotting experiments and found that compared with the control group, GCCs with overexpressed PTX3 had significantly lower expression of IL-4 and IL-10 at transcription (Fig. 5A, B) and protein levels (Fig. 5C, D), suggesting that PTX3 could negatively regulate the expression of IL-4 and IL-10. We further explored the relevant pathways of PTX3 using the KEGG pathway database and speculated that the JNK pathway might be the relevant pathway. Therefore, we performed western blotting assays and discovered that the upregulation of PTX3 in both the BGC-823 and SGC-7901 cell lines had no significant changes in the expression of JNK1/2, while the expression of phosphorylated JNK1/2 decreased notably (Fig. 5C, D). These results indicated that PTX3 decreased macrophage polarization to the M2-like phenotype via inhibiting the expression of IL-4 and IL-10 through negative regulation of JNK1/2 phosphorylation in GCCs.

### PTX3 suppresses the expression of LGR5 and M2 polarization of macrophages in milky spots *in vivo*

**Figure 6:**
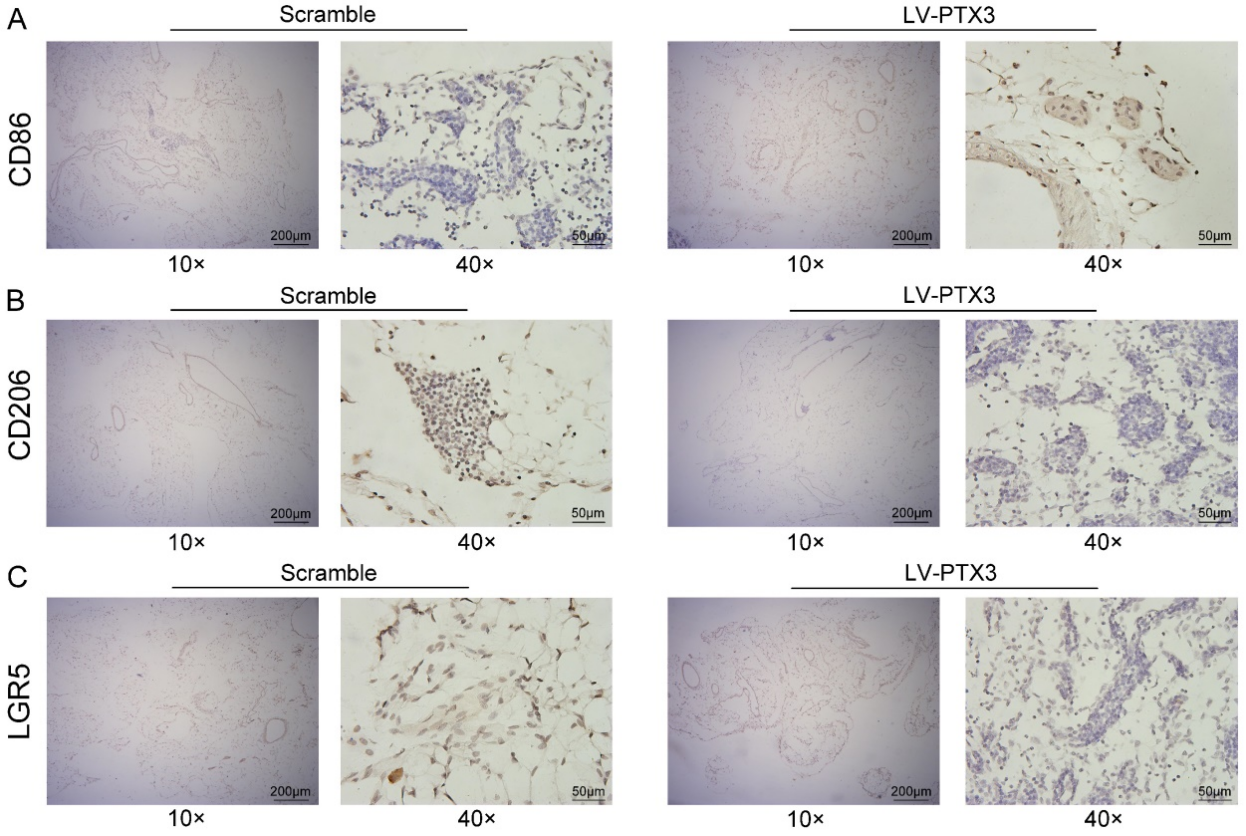
PTX3 suppresses the expression of LGR5 and M2 polarization of macrophages in milky spots *in vivo*. (A, B, C) Representative images of CD86, CD206 and LGR5 expression level of greater omentum were shown by immunohistochemistry (IHC) between the groups of Scramble and LV-PTX3 (Scale bars: 200 μm and 50 μm).

To further explore the impact of PTX3 on milky spots metastasis of GC, we transfected 615 murine GC cells (MFCs) with lentivirus vectors comprising overexpressed PTX3 (LV-PTX3) and the negative control (Scramble). Then the GCCs were injected intraperitoneally into 615 mice to promote the metastasis of GC into milky spots. Omental samples were collected on the 14th days respectively. Then we performed an IHC test on the greater omentum tissues to observe the expression of macrophages and discovered the higher CD86 expression while lower CD206 expression in the mice group that with PTX3 overexpression of gastric cancer cells compared with the control group (Fig. 6A, B), indicating that the up-regulation of the PTX3 in MFCs could inhibit the polarization to the M2 macrophages in the milky spot. Furthermore, we also tested the expression of the GC stem cell marker (LGR5) and found that the upregulation of PTX3 in MFCs decreased the expression of LGR5 in the milky spots metastasis of GC (Fig. 6C).

## Discussion

PTX3 is an important part of innate immunity and plays an important role in resisting certain microorganisms and regulating inflammation 24. In our previous research, we have proved that PTX3 is highly expressed in normal gastric mucosal epithelial cells and adjacent cancer tissues via qRT-PCR and western blot 17. To accurately verify the differential expression of PTX3, we further performed IHC tests consistent with previous studies. Because EMT is a mechanism of tumor metastasis progression 25, increasing evidence shows that EMT and CSC phenotypes are largely related. Furthermore, the prognosis of GC patients with detectable circulating tumor cells that express CSC markers is worse than that of GC patients without CSC markers 26. Having demonstrated that PTX3 can regulate the EMT process of GCCs 17, we further explored the effect of PTX3 on the stemness of GCCs in the present study. The results showed that the upregulation of PTX3 decreased stemness and inhibited the ability to form spheres in the GCCs.

During the process of peritoneal metastasis of GC, free tumor cells have been proved to be preferentially transplanted in the milky spots, which causes peritoneal dissemination and forms micrometastasis 15. The impact of milky spots on peritoneal metastasis is profound and pivotal in investigating the related mechanism of milky spot metastasis. The milky spot is a small specific structure composed of large macrophages. We believed that exploring the relative polarization of macrophages in the milky spots is the key to understanding the mechanism of milky spots in tumor metastasis. More evidence shows that TAMs are closely related to tumor growth, invasion and metastasis 27. M2 macrophage polarization promotes tumor progress; whereas M1-like polarized TAMs acts as tumor suppressors 28. Moreover, numerous studies have demonstrated a link between cancer-associated EMT and TAMs 29, 30. Because PTX3 could affect the EMT process and stemness in GCCs, we speculated that PTX3 is related to macrophage polarization. Through the IHC experiment, we demonstrated preliminarily that in tissues with low expressed PTX3, there was a higher density of M2 macrophages and a lower density of M1 macrophages. However, tissues with high expression of PTX3 showed contrary results. Furthermore, after adding recombinant PTX3 to the polarization process of M2 macrophages, the results indicated that rhPTX3 inhibited the polarization of M2 macrophages by reducing the expression of CD206 and the mRNA levels of the M2 marker. In addition, rhPTX3 suppressed the migration and invasion of GCCs promoted by M2 macrophages.

TME is essential for tumor metastasis and colonization. TAMs are key players in the TME that can promote tumor cell migration and invasion, matrix degradation and angiogenesis 6. In the process of GC peritoneal metastasis, GCCs preferentially colonize the milky spots and form metastatic nodules 31-33. The macrophages located in these nodules promote the growth of GCCs and create a chronic inflammation microenvironment 34. To explore the connection between PTX3 and milky spots, we co-cultured GCCs and macrophages *in vitro* to mimic the microenvironment of GC metastasis into milky spots. The results from the co-cultured system suggested that macrophages in PTX3 overexpressed group showed high expression of CD86 and low expression of CD206; consistent results were obtained on the transcription level. More importantly, we transfected PTX3 overexpressed lentivirus into mouse-derived GCCs (MFCs) and then injected intraperitoneally to further observe the characteristics of milky spots metastasis. Although the results are not particularly obvious, we found that compared with the control group mice (Scramble), the overexpression of PTX3 in MFCs promoted the expression of M1 macrophages but reduced the expression of M2 in milky spots of greater omentum. Moreover, several studies have indicated that GCCs selectively infiltrate into the milky spots in the early stages of peritoneal cancer dissemination 15. According to the cancer stem cells theory, tumor growth and metastasis are driven by cancer stem cells 35. Furthermore, results from previous studies pointed out that milky spots become a highly efficient “natural filter” for screening GC stem cells 36. Based on these views, we performed IHC tests of the milky spots to observe the expression of LGR5, a stemness marker of GC, and found that overexpression of PTX3 reduced the expression of LGR5, which we speculated lead to the inhibition of colonization of GCCs into milky spots.

In the TME, tumor cells excrete a substantial number of cytokines, such as IL-4 and IL-10, which could facilitate the polarization of M2 macrophages 37. As a potential tumor activator, IL-4 has been considered to promote the polarization of the M2 phenotype, thereby further inducing tumor metastasis 38. In addition, IL-10, recognized as an immunosuppressive cytokine, could boost carcinogenesis by hindering the production of IFN-γ and assisting tumors to escape immune responses. In some cancers, IL-10 is secreted by tumor cells and facilitates M2 phenotype differentiation in TAMs 28, 39. In the present study, we explored the function of PTX3 in the cytokines in human GCCs. We found that the expression of IL-4 and IL-10 were restrained in PTX3 overexpressed GCCs. Moreover, we analyzed relevant pathways through KEGG and found there was no overt change in JNK1/2 expression, while the expression of phosphorylated JNK1/2 decreased significantly in PTX3 overexpressed cells. Therefore, we concluded that PTX3 suppresses M2 macrophage polarization by depressing the secretion of IL-4 and IL-10 through negative regulation of JNK1/2 in GCCs.

## Conclusion

Our experiments demonstrated that PTX3 inhibited the stemness of GCCs. It also revealed a new function of PTX3 in promoting the alternative activation of TAMs in which PTX3 suppressed the differentiation of M2 macrophages by decreasing the expression of IL-4 and IL-10 via the JNK1/2 pathway. Furthermore, PTX3 inhibited GCCs metastasis into milky spots by attenuating the polarization of the M2 phenotype and reducing cancer cell stemness in milky spots. These findings suggested the biological role of PTX3 in the progression of GC, indicating an investigational basis for its usage as a tumor biomarker and may be potentially used as a target for the treatment of GC.

## Supplementary Material

Supplementary table.

## Figures and Tables

**Table 1:** Gene sequences for qRT-PCR

Gene	Sequences
PTX3	F, 5'- GTGTGGGTGGTGGCTTTGAT-3';
	R, 5'- CTCCCCACCCAACAATATTCC-3'
GAPDH	F, 5'- CCACTCCTCCACCTTTGAC-3';
	R, 5'- ACCCTGTTGCTGTAGCCA-3'
IL1β	F, 5'- CTGAGCACCTTCTTTCCCTTCA-3';
	R, 5'- TGGACCAGACATCACCAAGCT-3'
TNF-α	F, 5'- TGTAGCCCATGTTGTAGCAAACC-3';
	R, 5'- GAGGACCTGGGAGTAGATGAGGTA-3'
CXCL9	F, 5'- CAGCACCAACCAAGGGACTATC-3';
	R, 5'- TTCCTTCACATCTGCTGAATCTG-3'
CCR7	F, 5'- GCTCCAGGCACGCAACTTT-3';
	R, 5'- AGCTCACAGGTGCTACTGGTGAT-3'
Arg1	F, 5'- CAGTCGTGGGAGGTCTGACATAC-3';
	R, 5'- CTGCTGTGTTCACTGTTCGAGTT-3'
IL10	F, 5'- ACATCAAGGCGCATGTGAACT-3';
	R, 5'- TGCCTTTCTCTTGGAGCTTATTAAA-3'
TGF-β	F, 5'-CGCCAGAGTGGTTATCTTTTGA-3';
	R, 5'- CGGTAGTGAACCCGTTGATGT-3'
CCL22	F, 5'-ATGGATTGCCTGAGCCTG-3';
	R, 5'-CCTTTGTGGTCCCATATTCTGTC-3'
IL4	F, 5'-CCGTAACAGACATCTTTGCTGCC-3';
	R, 5'-GAGTGTCCTTCTCATGGTGGCT-3'
