# Supplementary material for: Pentraxin-3 inhibits milky spots metastasis of gastric cancer by inhibiting M2 macrophage polarization
Source: J Cancer. 2021 Jun 4;12(15):4686–97. doi: 10.7150/jca.58698 (PMC8210545; doi:10.7150/jca.58698)
Supplement: Supplementary file 1 — Supplementary table. [file jcav12p4686s1.pdf]

Detailed clinic parameters

Tissue code	Gender	Age	ASA	Stage	T	N	Operation date	Pathological type	Distant tumor metastasis
D09A1007	Male	65	1	II	2	1	2012/5/8	Adenocarcinoma	No
D09A1095	Male	55	2	II	1	2	2012/6/28	Adenocarcinoma	No
D09A1109	Female	71	1	I	1	1	2006/10/19	Adenocarcinoma	No
D09A1528	Female	44	1	II	2	1	2009/12/16	Adenocarcinoma	No
D09A1592	Male	58	1	I	1	1	2006/4/3	Adenocarcinoma	No
D09A1613	Male	53	1	I	1	0	2006/4/4	Adenocarcinoma	No
D09A1738	Male	55	2	I	1	1	2012/6/28	Adenocarcinoma	No
D09A1849	Female	54	2	II	3	1	2006/11/29	Adenocarcinoma	No
D09A1854	Male	55	1	I	1	0	2005/9/19	Adenocarcinoma	No
D09A1869	Male	51	1	II	2	1	2006/5/8	Adenocarcinoma	No
D09A1911	Male	63	1	I	1	0	2007/11/27	Adenocarcinoma	No
D09A2053	Female	63	2	II	1	2	2007/4/5	Adenocarcinoma	No
D09A2429	Female	50	1	II	3	1	2006/10/11	Adenocarcinoma	No
4101A5 005	Female	30	1	I	1	0	2006/4/25	Adenocarcinoma	No
4101A5 007	Male	66	2	I	1	2	2006/10/19	Adenocarcinoma	No
D09A3962	Male	73	1	II	2	1	2006/10/10	Adenocarcinoma	No
D09A4016	Female	51	1	II	1	2	2011/4/17	Adenocarcinoma	No
3601B2 013	Female	62	2	II	1	2	2005/11/21	Adenocarcinoma	No
D09A1618	Male	63	1	I	1	1	2011/5/12	Adenocarcinoma	No
3601B2 012	Male	42	1	I	1	1	2007/3/21	Adenocarcinoma	No
3205A3 005	Male	36	1	I	1	1	2005/9/23	Adenocarcinoma	No
3205A3 006	Female	63	2	I	1	0	2007/4/5	Adenocarcinoma	No
D09A1092	Male	54	1	I	1	0	2009/10/19	Adenocarcinoma	No
D09A1609	Female	50	1	II	3	1	2006/10/11	Adenocarcinoma	No
D09A1621	Male	62	2	II	1	2	2011/9/26	Adenocarcinoma	No
D09A1612	Male	75	2	II	3	0	2005/10/12	Adenocarcinoma	No
D09A1702	Male	55	1	I	1	1	2007/3/21	Adenocarcinoma	No
D09A1878	Male	53	1	I	2	0	2006/4/4	Adenocarcinoma	No
D09A1925	Male	64	1	I	1	1	2009/12/16	Adenocarcinoma	No

D09A2051	Female	30	1	II	1	2	2006/4/25	Adenocarcinoma	No
D09A2411	Male	70	1	I	1	0	2006/11/16	Adenocarcinoma	No
D09A2432	Female	55	1	II	2	2	2010/9/26	Adenocarcinoma	No
D09A2468	Female	70	1	II	3	1	2007/3/12	Adenocarcinoma	No
D09A2472	Female	64	2	I	1	1	2006/10/8	Adenocarcinoma	No
3205A3 007	Female	55	1	II	3	1	2010/4/5	Adenocarcinoma	No
3701A9 002	Male	72	2	I	1	1	2006/10/11	Adenocarcinoma	No
D09A1411	Female	59	2	II	2	2	2011/6/16	Adenocarcinoma	No
D09A1917	Male	63	1	II	1	2	2011/5/12	Adenocarcinoma	No
D09A1937	Male	65	2	II	3	1	2005/10/12	Adenocarcinoma	No
D09A1953	Male	63	2	I	1	1	2012/5/4	Adenocarcinoma	No
D09A6065	Male	60	2	I	1	1	2006/7/26	Adenocarcinoma	No
D09A6059	Female	55	2	II	2	2	2012/4/17	Adenocarcinoma	No
D09A1155	Male	60	1	I	1	1	2011/6/24	Adenocarcinoma	No
D09A2047	Male	64	1	I	1	1	2010/4/12	Adenocarcinoma	No
D09A2056	Male	58	1	II	1	2	2011/10/12	Adenocarcinoma	No
D09A2393	Male	62	1	I	1	1	2006/4/3	Adenocarcinoma	No
D09A2399	Female	77	1	II	3	1	2007/3/12	Adenocarcinoma	No
D09A2413	Female	62	2	I	1	1	2005/11/21	Adenocarcinoma	No
D09A0969	Female	33	1	II	1	2	2010/4/15	Adenocarcinoma	No
D09A1620	Male	63	1	II	1	2	2011/10/12	Adenocarcinoma	No
D09A1861	Male	66	2	II	2	2	2006/10/19	Adenocarcinoma	No
D09A1003	Female	59	2	II	1	2	2011/6/16	Adenocarcinoma	No
D09A6063	Male	71	2	I	1	1	2006/4/10	Adenocarcinoma	No
D09A1410	Male	65	2	I	1	1	2010/4/12	Adenocarcinoma	No
D09A1436	Female	55	2	I	1	1	2012/4/17	Adenocarcinoma	No
D09A1627	Male	63	1	I	1	1	2011/10/12	Adenocarcinoma	No
D09A1628	Male	60	2	II	2	1	2006/7/26	Adenocarcinoma	No
D09A6058	Male	64	1	I	1	1	2010/4/12	Adenocarcinoma	No
D09A2277	Male	58	1	I	1	1	2012/1/11	Adenocarcinoma	No
D09A2465	Male	43	2	II	3	0	2010/4/15	Adenocarcinoma	No
